# AI in epilepsy neuroimaging

**DOI:** 10.1097/WCO.0000000000001465

**Published:** 2026-02-19

**Authors:** Sophie Adler, Konrad Wagstyl

**Affiliations:** a Developmental Neurosciences, UCL Great Ormond Street Institute of Child Health, University College London; b School of Biomedical Engineering & Imaging Sciences, King's College London; c Great Ormond Street Hospital for Children, Supportive partner of the ERN EpiCARE, London, UK

**Keywords:** artificial intelligence, epilepsy, magnetic resonance imaging, neuroimaging

## Abstract

**Purpose of review:**

Recent advances in the capabilities and usability of artificial intelligence (AI) architectures coupled with increased availability of neuroimaging datasets has fuelled a rapid expansion in AI applications to epilepsy neuroimaging. This review summarizes the main applications of AI in epilepsy neuroimaging and suggests future directions for the field.

**Recent findings:**

A range of different machine learning approaches, from multi-layer perceptrons to volumetric and graph-based convolutional neural networks, have been utilized for prediction of whether people will have epilepsy, detection of structural epilepsy lesions, localization of seizure onset zones, segmentation of resection cavities after epilepsy surgery as well as for image enhancement.

**Summary:**

AI in epilepsy neuroimaging research has primarily focussed on lesion detection and localization, with a number of open and validated tools now available for evaluation across diverse settings. Additional applications of AI in epilepsy neuroimaging are either at earlier stages of development or emerging as new challenges. As these tools and their supporting evidence mature, further work addressing the hurdles of clinical integration is required.

## INTRODUCTION

Epilepsy is a chronic neurological disorder affecting 1 in 100 people. Neuroimaging investigations are fundamental in the diagnosis and management of epilepsy, with the current standard-of-care involving the visual assessment of images. However, human visual analysis is time-consuming and performance is limited by the level of user expertise. Furthermore, given the expanding volume of available neuroimaging techniques, there is an increasing challenge to efficiently review and synthesize information derived from different sources. 

**Box 1:**
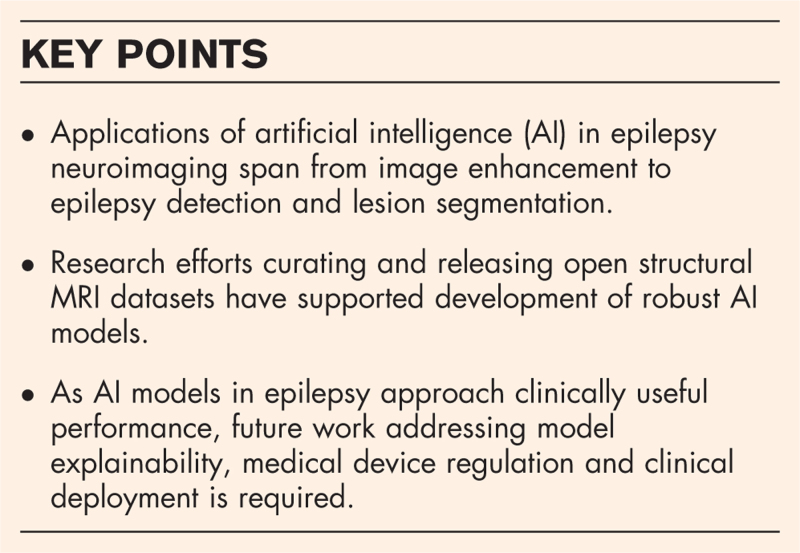
no caption available

Recent advances in artificial intelligence have led to an expansion of research aiming to couple neuroimaging data with deep-learning frameworks to answer clinically relevant and important questions. When appraising the relative strengths and limitations of AI approaches, useful questions to consider are:

(1) Training data: What data are used to train the model? Is the cohort representative of the relevant population? Is it real-world data or research quality data?
(2) Model task: What was the model being trained to do?
(3) Model architecture: What AI architecture was used? Does this have any inherent strengths or limitations?
(4) Model validation: How was the model validated? Did the researchers use cross-validation or have a separate test dataset?
(5) Model performance: What was the performance of the model? See Table 1 for an overview of commonly used performance metrics.

**Table 1:** Commonly used performance metrics to evaluate the performance of AI models

Metric	Task	Formula	Explanation
Accuracy	Classification	$\frac{TP+TN}{TP+TN+FP+FN}$	Measures the proportion of correctly classified instances among all instances.
Precision (positive predictive value, PPV)	Classification	$\frac{TP}{TP+FP}$	Measures how many of the positive predictions made by the model are actually correct.
Recall (sensitivity)	Classification	$\frac{TP}{TP+FN}$	Measures the proportion of actual positives that are correctly identified.
F1 score	Classification	$2\times \frac{\mathrm{Precision}\times \mathrm{Recall}}{\mathrm{Precision}\times \mathrm{Recall}}$	Providing a balanced measure of precision and recall for when classes are imbalanced.
Specificity	Classification	$\frac{TN}{TN+FP}$	Measures the proportion of actual negatives correctly identified.
AUC-ROC (area under the receiver operating characteristic curve)	Classification	-	Evaluates the ability of the model to distinguish between classes across different thresholds. A higher AUC indicates better discrimination.
IoU (intersection over union)	Image segmentation/object detection	$2\times \frac{\mathrm{Pre}d_{\mathrm{voxels}}\cap GT_{\mathrm{voxels}}}{\mathrm{Pre}d_{\mathrm{voxels}}\cup GT_{voxels}}$	Measures the overlap between predicted and actual object boundaries.
Dice coefficient	Image segmentation	$2\times \frac{\mathrm{Pre}d_{\mathrm{voxels}}\cap GT_{\mathrm{voxels}}}{\mathrm{Pre}d_{\mathrm{voxels}}+GT_{voxels}}$	Measures overlap in spatial similarity between a predicted segmentation and its ground truth

TP, true positives; TN, true negatives; FP, false positives; FN, false negatives; GT, ground truth; Pred, prediction.

**FIGURE 1:**
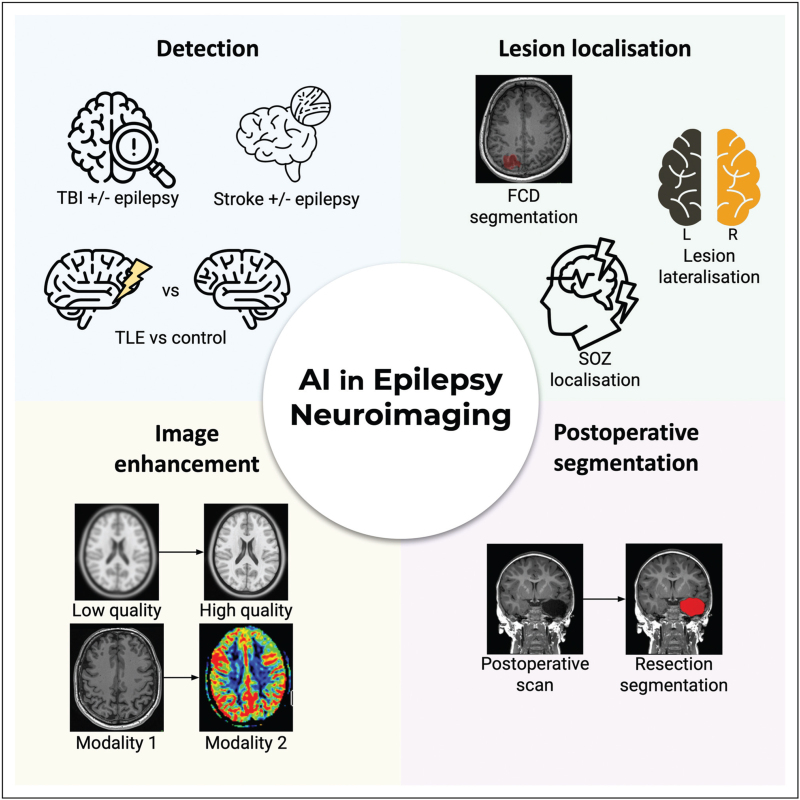
Overview of recent applications of AI in epilepsy neuroimaging.

This review describes the neuroimaging data commonly utilised, alongside specific recent applications of AI in epilepsy neuroimaging (Fig. 1).

### Epilepsy neuroimaging data for artificial intelligence

The International League Against Epilepsy (ILAE) advises that, if resources allow, an MRI scan, with the HARNESS-MRI protocol, should be performed after the first seizure [1]. The HARNESS-MRI protocol involves isotropic 3D T1-weighted and fluid-attenuated inversion recovery (FLAIR) sequences as well as 2D coronal T2-weighted images. Given that many epilepsy centres adhere to the ILAE neuroimaging guidelines [2], it is feasible to collate large retrospective datasets of these scans to train AI models either in a single large epilepsy centre [3], or through multi-centre initiatives [4,5,6^▪^]. In addition, a number of structural neuroimaging of epilepsy datasets have been made openly available including the Bonn Open Presurgery MRI dataset of people with epilepsy due to FCD [7^▪▪^], the Imaging Dataset for Epilepsy And Surgery (IDEAS) [8^▪▪^] and the Brain Imaging and Neurophysiology Database [9]. Obtaining ethical approval and / or appropriate consent, curating and annotating these datasets is a significant amount of work, but these resources provide invaluable training and / or independent validation data for the field. As a result, AI models for neuroimaging of epilepsy are most frequently trained on structural MRI data. However, there are also innovative AI approaches using diffusion weighted imaging (DWI), functional MRI, positron emission tomography (PET), high-field MRI data, MR fingerprinting or combinations of these.

### Artificial intelligence applications in epilepsy neuroimaging

*Segmentation of FCDs*. FCDs are malformations of cortical development associated with drug-resistant epilepsy. They are the second most common histopathological diagnosis in epilepsy surgery cohorts [10]. Automated methods for the detection of FCDs remains a research priority given that 31% of lesions are missed on visual analysis of MRI data by experts [11] and FCD is the underlying pathology in around 45% of patients who undergo epilepsy surgery without lesions visualized on their MRI scans [11,12^▪^,^13^].

A recent multi-centre study [14^▪▪^] compared a series of available AI approaches for FCD detection. This included MAP18 [15^▪▪^], which utilizes a neural network architecture trained on single voxels from T1 and morphometric feature maps; DeepFCD [16^▪▪^], a convolutional neural network trained on small patches of voxels from T1 and FLAIR; and MELD MLP [17], a multi-layer perceptron trained on surface-based features from T1 and FLAIR. During independent evaluation, F1 scores were as follows: 0.06 for deepFCD, 0.13 for MAP18, and 0.35 for MELD MLP. The ability of MELD MLP, the multi-layer perceptron developed by the Multi-centre Epilepsy Lesion Detection Project, to generalize to new datasets is further supported by additional independent validation studies [18,19] and is likely due to the large diversity in contributing hospitals and MRI scanners. The same study [14^▪▪^] also trained and evaluated a nnU-Net model [20^▪^] on a subset of their T1 and FLAIR images. nnU-Net is a self-configuring convolutional neural network which has been shown to perform well across a range of biomedical segmentation tasks. While this is not a fully independent validation, the 3D nnU-Net model provided the best F1 score (0.58). Chanra *et al.* similarly demonstrated good performance using a U-Net architecture, albeit on a small test dataset of 10 FCDs [21].

An ongoing challenge for the field has been the AI identification of additional, likely false positive, areas in addition to the FCD. MAP18 identified an average of 3.5 clusters per subject; MELD MLP 2.1 and DeepFCD 24.7 [14^▪▪^]. This results in the precision (i.e. the likelihood that an identified lesion is a true FCD) being low and is likely due to these approaches independently assessing only small portions of the brain. Approaches that allow models to incorporate context through considering a greater proportion of the brain have helped to address this challenge. The nnU-Net model, which divides the brain into patches of around 15% of MRI volume, predicted 0.9 clusters per subject. Similarly, the updated MELD Graph algorithm which receives features extracted across the whole cortical hemispheres [22^▪▪^] predicted a median of 0 false positive clusters per patient (IQR 0-0). It was also able to detect 64% of lesions previously missed by radiologists. As computer chips' (specifically graphics processing units') capacity to consider whole-brain context improves and neuroimaging datasets increase in size, we are likely to see further improvements in these metrics.

There has also been innovative research incorporating novel sequences into deep-learning frameworks [23–25]. Ding *et al.* [23] trained a nnU-Net model on established morphometric maps extracted from T1w images and novel T1 and T2 MR fingerprinting maps. While MacDonald-Laurs *et al.* incorporated PET features into the MELD surface-based framework [3]. These studies highlight the benefits of multi-modal integration for detecting subtle FCDs and reducing false-positive predictions. However, as these models were trained on single centre data, further work using larger multi-centre datasets capturing real-world heterogeneity in clinically acquired data will likely be required for these models to generalize well to data from new centres.

To date, a range of AI approaches for automated detection of FCDs have been developed, including multi-centre endeavours with open models and code, tested on independent validation cohorts. These AI tools, capable of detecting lesions that are often missed by conventional visual analysis, show promise as radiological diagnostic adjuncts. Future work is needed to move beyond research tools towards regulated medical devices that can be safely deployed at scale in epilepsy care.

### Diagnosis of temporal lobe epilepsy

Recent AI approaches utilizing multi-centre data have focused on temporal lobe epilepsy detection, differentiating patients from controls or other pathological groups, and temporal lobe epilepsy lateralization, determining whether the left or right side is affected. For the detection of temporal lobe epilepsy, Chang et al, employed a convolutional neural network to discriminate between a multicentre cohort of temporal lobe epilepsy patients (*n* = 157), Alzheimer's disease and healthy controls [26]. Using a larger multicentre cohort of patients with temporal lobe epilepsy (*n* = 1178), Gleichgerrecht *et al.* trained a convolutional neural network on T1w data to classify patients from controls with a sensitivity of 82% and specificity of 91%[27]. In a follow-on study, they used saliency methods to interrogate which parts of the MRI scan were important for determining disease status[6^▪^]. Interestingly, the saliency maps highlighted similar cortical and subcortical regions for visible lesions and MRI negative patients, suggesting that there are some common brain changes across the spectrum of lesional MRI-visibility.

The most common histopathological cause of temporal lobe epilepsy is hippocampal sclerosis (HS), which is missed on visual analysis of MRI scans in approximately 10% of cases [12^▪^,^13^]. For automated lateralization and detection of hippocampal sclerosis, Ripart *et al.*[28^▪▪^] used a deep-learning algorithm, Hippunfold [29], to segment the hippocampus and then trained a logistic regression classifier on hippocampal surface-based features. The model, AID-HS, was able to successfully lateralize 97% of patients, including 92% of patients considered MRI negative, and to differentiate HS patients from controls and FCD disease controls in 91%. It has since been independently validated [30] in a study that additionally introduced a support vector classifier model trained on T1 and FLAIR hippocampal features.

### Prediction of epilepsy

Tumours and traumatic brain injury are conditions frequently associated with seizures [31,32], where accurate prediction of whether patients will develop seizures could affect treatment pathways. In low-grade gliomas, the prevalence of epilepsy varies with patient age and tumour histology. Radiomics is an evolving field where shape, intensity, texture and location features are extracted from MRI data. Recent work has applied machine-learning methods to radiomics features to predict tumour-related epilepsy with classification accuracies (AUCs) ranging between 0.88 and 0.97 [33–35]. To predict likelihood of post traumatic epilepsy, Akrami *et al.* combined lesion volumes and functional MRI derived metrics into a machine-learning framework and obtained an AUC-ROC of 0.78 [36]. Although encouraging performance is reported in the prediction of tumour-related and post-traumatic epilepsy, validation on independent cohorts is required to fully elucidate how generalizable these models are.

### Localization of the seizure onset zone

The seizure onset zone (SOZ) is the cerebral region responsible for generating the seizure and its localization is integral for epilepsy surgery planning. To date, the gold-standard for defining the SOZ is stereoelectroencephalography (sEEG). However, there has been recent work applying machine-learning frameworks to MRI data to determine the SOZ. One study combining resting-state functional MRI connectomics, expert knowledge and deep learning demonstrated a SOZ localization F1 score of 92% [37]. While another group applied machine-learning models to 23Na-MRI data acquired at 7T to generate SOZ priors, and demonstrated that when incorporated with existing structural and functional priors, the 23Na-MRI data improves estimation of the SOZ [38]. More precise estimation of the SOZ is likely to involve multimodal integration of electrophysiology data with structural and functional MRI data.

### Segmentation of epilepsy surgery resection cavities

For research investigating outcomes after epilepsy surgery, accurate segmentation of resection cavities from post-operative MRI scans is often required in order to establish exactly what tissue and white matter tracts have been resected. Courtney and colleagues compared four automated tools for the segmentation of resection cavities, and found that the tissue-based classification models (Epic-CHOP and ResectVol) performed better than the deep-learning based (Resseg and Deep Resection), segmenting 84% and 88%, compared to 44% and 46%, of resection cavities respectively [39^▪^]. More recently, the RAMPS pipeline that utilizes deep-learning for skull stripping and brain parcellation as well as more classical tissue classification and subtraction methods has been shown to outperform existing methods [40^▪^]. Future work, utilizing these AI derived segmentations of surgical resections, is likely to yield important insights into what grey and white matter disruption is required or needs to be avoided for optimal post-surgical outcomes both for seizure freedom and neuropsychological function.

### Image enhancement and synthesis

An interesting application of AI in epilepsy neuroimaging has been to either enhance existing MRI data or create synthetic images. For example, one challenge with high field MRI data is artefacts due to magnetic field inhomogeneities. For 7T FLAIR scans, temporal lobe regions are particularly affected. Using a voxel-wise neural network, Uher *et al.* generated synthetic FLAIR-like images with improved signal and reduced contrast attenuation in MRI-negative epilepsy patients. Alternatively, generative adversarial networks have been employed to synthesize Single-Photon Emission Computed Tomography (SPECT) images from MRI or PET data [41] and PET images from Arterial Spin Labelling (ASL) data [42]. Synthetic SPECT and PET data is an exciting prospect given that it could reduce or remove exposure to radiation currently required for PET/SPECT investigations.

In many low and middle-income countries, there is scarce availability of high-field (1.5 T, 3 T or 7 T) MRI scanners. Low-field (<1 T) MRI is more widely available and proof-of-concept work suggests it can be used for epilepsy lesion detection [43^▪^]. However, it has lower signal-to-noise ratios. Promising work has demonstrated utilizing deep-learning to improve the image quality of low-field data and the visibility of epilepsy lesions [44], which has the potential to be transformative in democratizing epilepsy diagnosis in resource-limited settings.

### Future directions

**FIGURE 2:**
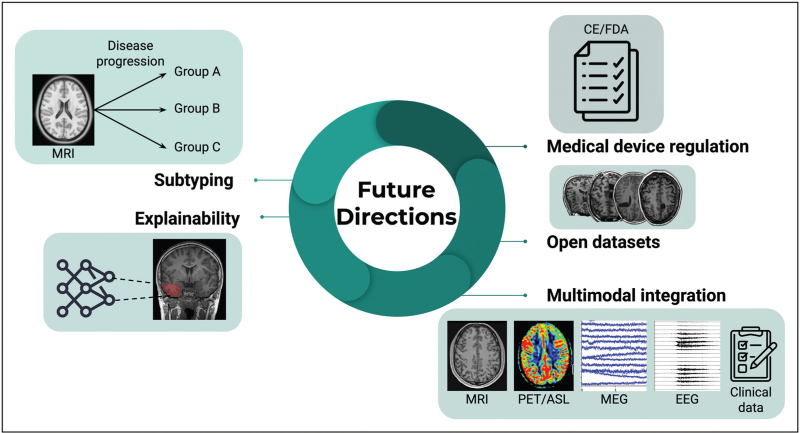
Future directions for AI in epilepsy neuroimaging.

AI in Epilepsy neuroimaging is an exciting field of rapid innovation, with a number of research areas primed for future development (Fig. 2).

First, data-driven stratification of patient cohorts into subgroups using AI approaches might reveal multiple opportunities for precision medicine. Examples of this include computational subtyping of patients by their estimated disease progression patterns [45,46], models which incorporate neuroimaging-derived features to predict post-surgical outcome [47] and automated characterization of epilepsy lesions into their histopathological subtypes [22^▪▪^,^48^]. While parallel advances in data-driven molecular diagnostics may provide more robust labels on which future MRI classification algorithms can be trained [49–51]. Second, there is significant scope for further research incorporating multi-modal data both within neuroimaging i.e. integration of PET, ASL and structural MRI data, as well as including other modalities e.g. genetic, clinical and electrophysiological data. Third, recent advances in multimodal large-language medical models such as MedGEMMA [52] have not yet been specifically extended to epilepsy, but this is likely to become an active research area. Last, as AI models approach clinically useful performance in epilepsy, a number of other considerations will become increasingly central, including model explainability, how to regulate models as medical devices and the practical challenges of deploying AI tools in hospital workflows [53–55].

## CONCLUSION

The incorporation of AI into Epilepsy neuroimaging is advancing research across a range of applications. Fundamental to the development and appraisal of research involving AI is having an advanced understanding of both the clinically useful questions to address and the AI specifics which include the training and test data, the machine learning task and the reported evaluation metrics. Progress has been and will continue to be accelerated by open datasets, code and models, that facilitate rapid innovation and independent validation of new advances. Current clinical translation of AI in epilepsy neuroimaging is modest, but in the near future we will likely see it impact at multiple stages throughout the patient journey, from first diagnosis and treatment planning to post-surgical follow-up. As a result these advances will become incorporated into the practice of a range of healthcare professionals, including family doctors, epilepsy nurses, neurologists, neuroradiologists and neurosurgeons.

## Acknowledgements

*We thank Jieun Seo and Dr Mathilde Ripart for revising the manuscript and generating the figures. All research at UCL Great Ormond Street Institute of Child Health is made possible by the NIHR Great Ormond Street Hospital Biomedical Research Centre. The views expressed are those of the author(s) and not necessarily those of the NHS, the NIHR or the Department of Health. This work was additionally supported by ERN EpiCARE.*

### Financial support and sponsorship

*None.*

### Conflicts of interest

*There are no conflicts of interest.*

## REFERENCES AND RECOMMENDED READING

Papers of particular interest, published within the annual period of review, have been highlighted as:

▪ of special interest
▪▪ of outstanding interest
